# Identification of *Sr67*, a new gene for stem rust resistance in KU168-2 located close to the *Sr13* locus in wheat

**DOI:** 10.1007/s00122-023-04530-8

**Published:** 2024-01-24

**Authors:** Jyoti Saini Sharma, Mingzhe Che, Thomas Fetch, Brent D. McCallum, Steven S. Xu, Colin W. Hiebert

**Affiliations:** 1 Agriculture and Agri-Food Canada, Morden Research and Development Centre, 101 Route 100, Morden, MB R6M 1Y5 Canada; 2 Present Address: Department of Plant Pathology, University of Minnesota, Saint Paul, MN 55108 USA; 3 Department of Plant Pathology, China Agricultural University, Beijing, 100193 People’s Republic of China; 4 Crop Improvement and Genetics Research Unit, Western Regional Research Center, USDA-ARS, 800 Buchanan Street, Albany, CA 94710 USA

## Abstract

**Key message:**

*Sr67* is a new stem rust resistance gene that represents a new resource for breeding stem rust resistant wheat cultivars

**Abstract:**

Re-appearance of stem rust disease, caused by the fungal pathogen *Puccinia graminis* f. sp. *tritici* (*Pgt*), in different parts of Europe emphasized the need to develop wheat varieties with effective resistance to local *Pgt* populations and exotic threats. A Kyoto University wheat (*Triticum aestivum* L.) accession KU168-2 was reported to carry good resistance to leaf and stem rust. To identify the genomic region associated with the KU168-2 stem rust resistance, a genetic study was conducted using a doubled haploid (DH) population from the cross RL6071 × KU168-2. The DH population was phenotyped with three *Pgt* races (TTKSK, TPMKC, and QTHSF) and genotyped using the Illumina 90 K wheat SNP array. Linkage mapping showed the resistance to all three *Pgt* races was conferred by a single stem rust resistance (Sr) gene on chromosome arm 6AL, associated with *Sr13*. Presently, four *Sr13* resistance alleles have been reported. *Sr13* allele-specific KASP and STARP markers, and sequencing markers all showed null alleles in KU168-2. KU168-2 showed a unique combination of seedling infection types for five *Pgt* races (TTKSK, QTHSF, RCRSF, TMRTF, and TPMKC) compared to *Sr13* alleles. The phenotypic uniqueness of the stem rust resistance gene in KU168-2 and null alleles for *Sr13* allele-specific markers showed the resistance was conferred by a new gene, designated *Sr67*. Since *Sr13* is less effective in hexaploid background, *Sr67* will be a good source of stem rust resistance in bread wheat breeding programs.

**Supplementary Information:**

The online version contains supplementary material available at 10.1007/s00122-023-04530-8.

## Introduction

Wheat stem rust, caused by infection with the biotrophic fungal pathogen *Puccinia graminis* Pers.:Pers. f. sp*. tritici* Eriks & E. Henn (*Pgt*), is a disease of high priority on the Canadian Prairies and other major global wheat producing regions. While stem rust can be controlled by resistance (Sr) genes, recent experience shows the risk posed by the evolution of virulence in the pathogen population. Following the detection of the highly virulent *Pgt* race TTKSK (commonly known as Ug99) in 1998, 15 variants have been detected in several East African countries, Yemen, Iran, and Iraq (http://rusttracker.cimmyt.org; Fetch et al. 2016; Patpour et al. 2016; Pretorius et al. 2000; Pretorious et al. 2012; Terefe et al. 2016; Nazari et al. 2009; Nazari et al. 2021). Additionally, since 2012 other highly virulent, unrelated races (TRTTF, TKTTF, and TTRTF) were detected in different European countries (Patpour et al. 2022). The re-emergence of stem rust draws the attention of wheat researchers to look for an effective approach to avert future epidemics.

Olivera et al. (2022) reported detection of a unique isolate of *Pgt* race (TKHBK) from fields surrounded by barberry plants, the alternate host of *Pgt,* in Spain. This race has virulence for resistance genes *Sr31*, *Sr33*, *Sr53* and *Sr59*, but is avirulent to the nearly universally susceptible durum line Rusty, which was used to develop multiple mapping populations. Villegas et al. (2022) suggested the likely involvement of barberry in the sexual cycle of *Pgt* in Spain and hence the likelihood of unique races. With the emergence of new races and dispersal of known races, it is important to incorporate new and effective Sr genes into breeding programs. To search for novel Sr genes, the foremost step is phenotypic characterization of available genetic resources with the most threatening local, where possible exotic *Pgt* races.

Kyoto University wheat accession KU168-2 (*Triticum aestivum*) that originated from the Inner Mongolia Autonomous Region in China has resistance to leaf rust (caused by *P. triticina* and stem rust (Tanaka 1983; Che et al. 2019). Che et al. (2019) found that KU168-2 carried leaf rust resistance genes *Lr33*, *Lr34* and a seedling resistance gene on chromosome arm 6AL; however, the genetic basis for stem rust resistance is unknown. The purpose of this study was to determine the inheritance of the stem rust resistance in KU168-2, genetically map the resistance, and compare the resistance to any known Sr gene with a similar map position.

## Material and methods

### Plant materials

A doubled haploid (DH) population (*n* = 110) was developed from a cross between the susceptible wheat line RL6071 (Prelude/8 * Marquis * 2/3/Prelude//Prelude/8 * Marquis) and KU168-2 using the maize pollination method (Che et al. 2019). Both RL6071 and KU168-2 are hexaploid wheat lines. Tetraploid wheat reference single-gene lines carrying each known allele of *Sr13*, namely Rusty-Kl-B (*Sr13a*), Rusty-14803 (*Sr13b*), Rusty-ST464-C1 (*Sr13c*), and CAT-A1 (*Sr13d*), (Gill et al. 2021; Zhang et al. 2017) along with hexaploid line Sr13a (Knott 1990), were used in multi-race stem rust comparisons with KU168-2 and a subset of DH lines.

### Phenotyping with* Pgt*

Stem rust seedling assays were performed on the parents (RL6071 and KU168-2), DH population, and lines carrying the different *Sr13* resistance alleles. The inoculation of plant materials was carried out as described by Hiebert et al. (2011). Isolates of *Pgt* races TTKSK (SA13), TPMKC (W1373) and QTHSF (W1347) were used to phenotype the DH population. Additional phenotyping was done with a total of five races TTKSK, TPMKC, QTHSF, RCRSF (W001), and TMRTF (W1311) to compare the gene discovered in KU168-2 with *Sr13* using 10 resistant (R) and 10 susceptible (S) lines from the DH population based on the TTKSK score, and the reference lines carrying the *Sr13* alleles. After inoculation, plants were kept for 20–22 h at 18 °C in dark mist chambers with 100% relative humidity. The next day after a brief drying period, plants were moved to a greenhouse [22 ± 3 °C with a 16/8 h (day/night)]. To further compare the Sr gene from KU168-2 with alleles of *Sr13*, the above lines were also phenotyped with races RCRSF and TMRTF while placing the plants in a growth cabinet with temperatures of 18 °C under light and 15 °C during darkness as *Sr13* reported to be temperature-sensitive (Roelfs and McVey 1979).

Plants were scored 13–14 days after inoculation using a 0–4 (0,;, 1–4) rating scale with additional “ + ” and “−” to indicate larger or smaller pustules for a given infection type (IT) (Stakman et al. 1962). Plants with IT ranging from 0 to 2 were classified resistant (R) whereas IT ranging from 3 to 4 were classified as susceptible (S). For initial QTL mapping, the IT score was converted to a linearized infection type (LIT) and rounded to a 0 to 9 scale (Zhang et al. 2014). For genetic mapping of the resistance as a qualitative trait, stem rust IT ratings for DH lines were classified as R and S as described above.

### Genotyping and linkage mapping

The DH population was genotyped with the wheat 90 K iSelect Infinium SNP array (Wang et al. 2014). SNP alleles were called and markers were filtered for polymorphism using GenomeStudio software (Illumina, San Diego, USA). To reduce redundant marker data, genotypic data were analyzed with the BIN function in QTL IciMapping Ver 4.1.0.0 software (Meng et al. 2015) to identify co-segregating markers. Data from a set of non-redundant SNP markers were inputted into MapDisto 1.8.2 software (Lorieux 2012) and were used to develop linkage maps as described by Che et al. (2019). The LIT score was used for the identification of QTL for resistance to the *Pgt* races TTKSK, TPMKC, and QTHSF. The QGENE (4.4.0) (Joehanes and Nelson 2008) software package was used for the QTL analysis. As the phenotypic ratio fitted neither a one nor a two gene model, the purpose of the QTL analysis was to determine if one or two genes were conferring resistance to stem rust in the population. Once the analysis indicated that a single gene was responsible for the resistance to multiple *Pgt* races used to phenotype the population, the phenotypic data were treated as a qualitative trait, a common procedure for mapping hypersensitive rust resistance genes in wheat.

Five SNP markers from the 90 K iSelect Infinium SNP array that were closely associated with the *Sr* gene on chromosome arm 6AL were converted to Kompetitive allele-specific PCR (KASP) markers (*Kwh290*, *Kwh291*, *Kwh292*, *Kwh293*, and *Kwh294*). The KASP genotyping procedure was done as described by Kassa et al. (2016). In addition, the gene-based marker, Sr13F/R Primer, reported by Zhang et al. (2017) was used to genotype the DH population and a check line carrying the *Sr13*a allele. PCR conditions were as described by Zhang et al. (2017), the amplified PCR products were digested with restriction enzyme *Hha*I and cleaved products were run on ethidium bromide-stained agarose gels. Four SNP-based semi-thermal asymmetric reverse PCR (STARP) markers, *rwgsnp37.2*, *rwgsnp38*, *rwgsnp39*, and *rwgsnp40*, reported to differentiate between the *Sr13* alleles were used to characterize the parental lines along with reference lines carrying each of the four *Sr13* alleles (Gill et al. 2021 and Saini et al. 2018). PCR protocols for STARP marker analysis followed Long et al. (2017) and samples were run on 6% non-denaturing polyacrylamide gels as in Saini et al. (2018). Genomic sequence analysis of the *Sr13*-CNL13 gene was performed by using the four primers 6ACNL13F7/R2, 6ACNL13F4/R7, 6ACNL13F3/R8, and 6ACNL13F5/R6 reported in the Zhang et al (2017). These primers covered the entire coding and intron sequences of the *Sr13*-CNL13 and were used to compare KU168-2 with the reference stocks for the *Sr13* alleles.

## Results

**Fig. 1:**
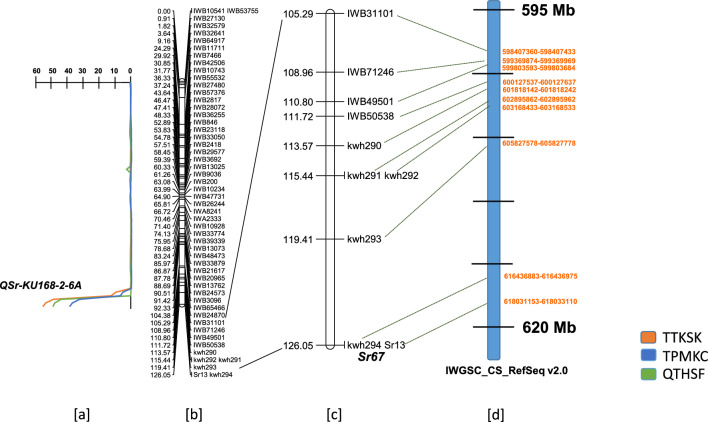
Genetic map of the chromosome 6A developed in the RL6071 × KU168-2 double haploid population **a** QTL peaks for resistance to *Pgt* races TTKSK, TPMKC, and QTHSF, **b** chromosome 6A linkage map, **c** the chromosome 6A region showed co-segregation of the *Sr13* gene-based marker with *Sr67*, **d** Mapping of the KASP and SNP marker sequences on the IWGSC 6A physical map

**Fig. 2:**
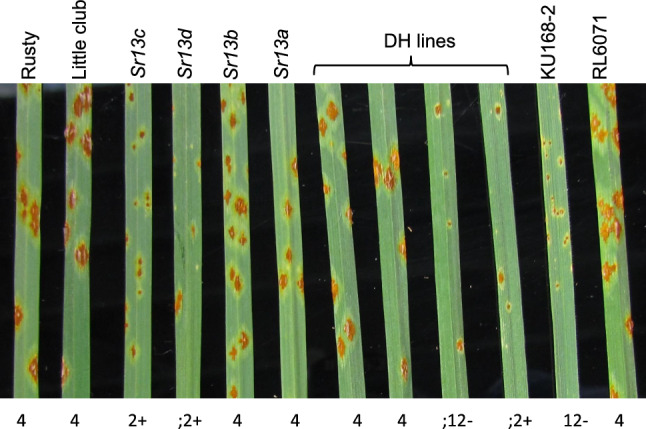
Phenotypic screening of lines carrying *Sr13* alleles, Rusty-Kl-B (*Sr13a*), Rusty-14803 (*Sr13b*), Rusty-ST464-C1 (*Sr13c*), and CAT-A1 (*Sr13d*) along with RL6071 × KU168-2 DH population parents, and two DH lines that lack *Sr67* and two DH lines that carry *Sr67* using *Pgt* race QTHSF

Phenotyping the RL6071 × KU168-2 DH population with *Pgt* races TTKSK, TPMKC, and QTHSF showed the phenotypic data fell between a one and a two gene ratio (Table 1); however, the resistance or susceptibility of each line corresponded across the three races, indicating that the same allele conferred resistance to all three races. QTL analysis revealed only one genetic region on the chromosome arm 6AL, *QSr-KU186-2-6A*, conferred resistance against all three *Pgt* races. The *QSr-KU168-2-6A* on chromosome arm 6AL was positioned at the end of the genetic map and flanked by marker *Kwh290* on one side and the peak LOD was at the terminal marker, *Kwh294* (Fig. 1). Since a single gene explained the observed resistance and the resistance gene is new (further evidence below), the gene was designated as *Sr67*. The *Sr67* region corresponded to the map position of *Sr13* based on 90 K iSelect Infinium SNP array markers and their coordinates on the International Wheat Genome Sequencing Consortium (IWGSC) ReqSeq v2.0 wheat reference genome. The LOD scores for the three *Pgt* races were between 30 and 60 and coefficients of variance (*R*^*2*^ × 100) ranged between 80 and 90% (Table 2). The five KASP markers developed in the current study, *Kwh290*, *Kwh291*, *Kwh292*, *Kwh293*, and *Kwh294* were mapped in the DH population (Table 3). The skewed phenotypic ratios for *Sr67* were mirrored by the ratios of the DNA markers that mapped distally on chromosome arm 6AL (Fig. 2). The resistance gene was mapped as a single, qualitative gene and corresponded to the region of chromosome arm 6AL defined by the QTL from the initial analysis. *Sr67* co-segregated with KASP marker *Kwh294*, and conferred resistance to races TTKSK, TPMKC, and QTHSF (Fig. 1).

**Table 1:** Responses of the RL6071 × KU168-2 double haploid population with three *Puccinia graminis* f. sp. *tritici* (*Pgt*) races

Race^a^	Observed^b^			One gene ratio (1:1)^c^		Two gene ratio (3:1)	
	R	S	Total	χ^*2*^	*P*	χ^*2*^	*P*
TTKSK	67	38	105	8.0	0.005	7.0	0.008
TPMKC	67	39	106	7.4	0.006	7.7	0.005
QTHSF	68	39	107	7.9	0.005	7.5	0.006

^a^Resistance to each race co-segregated

^b^R: Resistant, S: Susceptible

^c^χ^*2*^: Chi-squared symbol, *P*: Probability

**Table 2:** Quantitative trait loci (QTL) identified in the RL6071 × KU168-2 double haploid population against the *Puccinia graminis* f. sp. *tritici* (*Pgt*) races TTKSK, TPMKC, and QTHSF

*Pgt* race	QTL	Flanking marker	Position (cM)	LOD	Add.^a^	*R*^*2*^ × 100
TTKSK	*QSr.Ku168-2_6A*	*Kwh290-*	124	55.3	− 3.9	90.1
TPMKC	*QSr.Ku168-2_6A*	*Kwh290-*	124	38.0	− 3.9	79.6
QTHSF	*QSr.Ku168-2_6A*	*Kwh290-*	124	49.0	− 3.7	87.1

^a^Add. = Additive effect of the QTL, negative values indicate resistance derived from wheat accession KU168-2

**Table 3:** Kompetitive allele-specific (KASP) polymerase chain reaction (PCR) markers source, single nucleotide polymorphism (SNP) name, primer sequence information developed from the 90 K iSelect Infinium SNP array SNPs on the 6AL region in the RL6071 × KU168-2 double haploid population

Marker	Name	Primer	Primer A1 (5′-3′) sequence^a^
*Kwh290*	Tdurum_contig8166_2257	*Kwh290-A1* *Kwh290-A2* *Kwh290-C*	{Tail-1}CCGGGCGCAAAAAGGGCTC {Tail-2}ATCCGGGCGCAAAAAGGGCTT CTAGGCTGCTTGCTGCCTCGT
*Kwh291*	BS00029127_51	*Kwh291-A1* *Kwh291-A2* *Kwh291-C*	{Tail-1}GTTGTGCGGAAACAGATGCTTGATT {Tail-2}GTGCGGAAACAGATGCTTGATG GAGACGCTATTTATCGCACACCACA
*Kwh292*	BS00096240_51	*Kwh292-A1* *Kwh292-A2* *Kwh292-C*	{Tail-1}CACCTCGTGGAACCCGACG {Tail-2}GCACCTCGTGGAACCCGACA CCCTGCTGGCCTACTATGTCTGAT
*Kwh293*	wsnp_Ra_c11651_18855691	*Kwh293-A1* *Kwh293-A2* *Kwh293-C*	{Tail-1}GCCAGCCAGAAACTAACATATCG {Tail-2} GGCCAGCCAGAAACTAACATATCA GTAGTATTTATATATTGCACTCTTGTGGTT
*Kwh294*	GENE-4208_788	*Kwh294-A1* *Kwh294-A2* *Kwh294-C*	{Tail-1}CAACAAATAAACCACCAATCAACAATGATATT {Tail-2} ACAAATAAACCACCAATCAACAATGATATC ACTCTACTTGGATAATGAAACCACTTACAA

^a^Tail-1(FAM tail- GAAGGTGACCAAGTTCATGCT),Tail-2 (VIC tail- GAAGGTCGGAGTCAACGGATT)

**Fig. 3:**
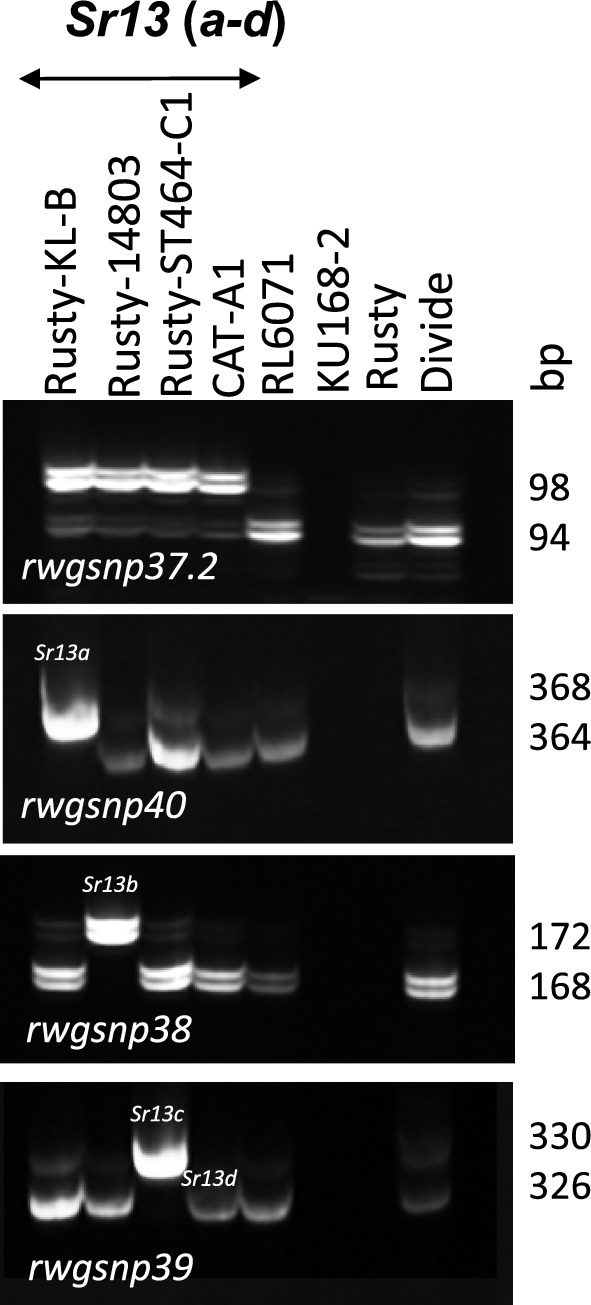
Genotyping lines with *Sr13* alleles, Rusty-Kl-B (*Sr13a*), Rusty-14803 (*Sr13b*), Rusty-ST464-C1 (*Sr13c*), and CAT-A1 (*Sr13d*) along with RL6071 and KU168-2 using SNP-based semi-thermal asymmetric reverse PCR (STARP) marker (*rwgsnp37.2*, *rwgsnp38*, *rwgsnp39*, and *rwgsnp40*)

**Fig. 4:**
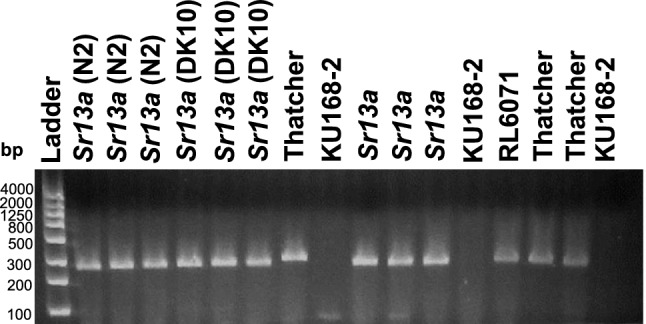
Comparing KU168-2 with *Sr13*-bearing stocks using gene-based Sr13F/R Primer reported by Zhang et al. (2017)

**Fig. 5:**
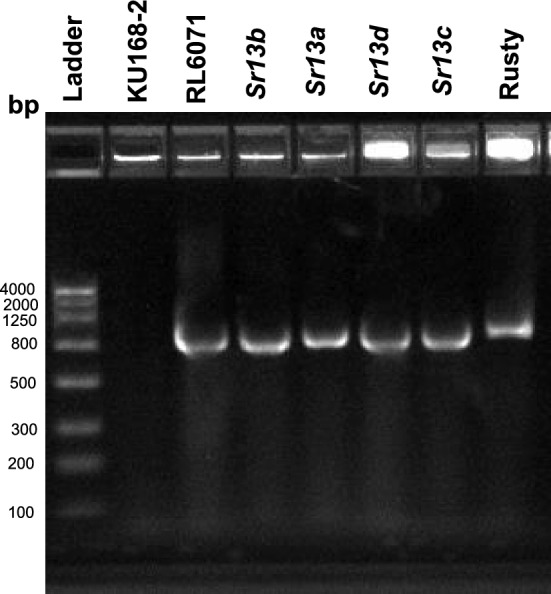
The *Sr13* gene sequence analysis with the primer 6ACNL13F4/R7 on the *Sr13* haplotypes R1-R4: Rusty-Kl-B, Rusty-14803, Rusty-ST464-C1, and CAT-A1 along with RL6071 and KU168-2

Tests of KU168-2 with KASP and STARP markers tightly linked to *Sr13* (*rwgsnp37.2*, *rwgsnp38*, *rwgsnp39*, and *rwgsnp40*) generated no amplification indicating null alleles (Fig. 3). The *Sr13* (CNL13) gene-based marker Sr13F/R Primer (Zheng et al. 2017) failed to amplify the resistance allele in the DH population, instead amplifying the susceptible allele present in RL6071 and a null allele in KU168-2 (Fig. 4). This polymorphism between the parental lines allowed Sr13F/R Primer to be mapped as a dominant (presence/absence) marker in the RL6071 × KU168-2 DH population and co-segregated with both the susceptibility allele and KASP marker *Kwh294* (Fig. 1). Again, there was no amplification in KU168-2 with the *Sr13* sequencing primers 6ACNL13F7/R2, 6ACNL13F4/R7, 6ACNL13F3/R8, and 6ACNL13F5/R6 (Fig. 5).

Multi-pathotype tests using five differentiating *Pgt* races on four *Sr13* resistance alleles durum cv. Rusty background, parental lines KU168-2 and RL6071, and sets of 10 DH lines with and without *Sr67* showed that KU and lines carrying *Sr67* were resistant whereas the lines with *Sr13* alleles showed differential reactions (Table 4; Fig. 2). The resistance to all races tested here co-segregated with *Sr67*. Race RCRSF was virulent to all alleles of *Sr13* while *Sr67* conferred resistance, thus differentiating the two genes based on phenotype (Supplemental Fig. 1A). Other races showed varying differences between *Sr67* and different alleles of *Sr13*, though some of those differences appeared to be temperature dependent (Table 4). As expected, *Sr13* ITs tended to be lower at higher temperatures. In contrast, *Sr67* generally showed a lower IT at lower temperatures (Table 4; Supplemental Fig. 1B).

**Table 4:** Comparison of responses conferred by *Sr67* and *Sr13* alleles using five *Pgt* races in different testing facilities

	Infection types^c^									
	DH Lines^b^				‘Rusty’ reference stocks					
*Pgt* Race (env.)^a^	*Sr67* +	*Sr67* -	KU168-2	RL6071	Rusty	*Sr13a*	*Sr13b*	*Sr13c*	*Sr13d*	LMPG-*Sr13a*
RCRSF (GH)	;1- to 12-	34 to 4	1–1	34	33 +	3 ±	33 +	3 ±	3–3	34
RCRSF (Cab)	;1- to 12-	3- to 34	;1-	3 ±	3 ±	33-	33-	33-	3 +	34
TMRTF (GH)	; to;2-	34 to 4	;1-	4	4	3 ±	2 ±	33-	3–3	33 +
TMRTF (Cab)	0; to;1	23 to 33 +	0	23	3–3	33-	2–2	2–2	33-	3-
TPMKC (GH)	11 + to 23-	4	22 +	4	4	12-	1-	1–1	11 +	12-
TPMKC (Cab)	;1- to 12-	3 ± to 34	12-	3–3	3 ±	3–3	11 +	2–2	–	3–3
QTHSF (GH)	;1- to;23	4	12-	33 +	–		4	2 +	;2 +	–
TTKSK (BL3)	0 to 0;	33 + to 34	0	34	–	11 +	11 +	2–2	3 +	–

^a^Race and isolate numbers were RCRSF (W0001), TMRTF (W1311), TPMKC (W1373), QTHSF (W1347), and TTKSK (SA31). Environments (env.) were GH: greenhouse, 22 °C ± 3; Cab, growth cabinet, 18 °C day and 15 °C night; BL3, biocontainment lab, 22 °C day and 18 °C night

^b^Ten DH lines each were classified as having *Sr67* (+) or lacking *Sr67* (−) with the range of ITs for each class presented

^c^Infection type as described by Stakman et al. (1962)

## Discussion

The ongoing need for new sources of stem rust resistance led to the assessment of germplasm collections in our possession. The strong stem rust resistance response of KU168-2 made it an ideal candidate for genetic analysis. Although the phenotypic ratio for resistant and susceptible DH lines deviated from the expected 1:1, linkage mapping showed that all resistant plants could be accounted for by a single allele (Table 1; Fig. 1). Segregation of the linked markers also showed abnormal segregation. Likewise, a leaf rust resistance gene identified in chromosome arm 6AL in the same population also showed abnormal segregation (Che et al. 2019). Assessment of the segregation of loci along chromosome 6A in the DH population showed that most of the chromosome fitted a 1:1 ratio, however, the distal region spanning 25 cM of the long arm, which included *Sr67*, showed skewed inheritance (*p* < 0.05) in favor of the KU168-2 alleles. Given that the DH population is derived solely from female gametes, there could be a few explanations for the skewed ratios observed, including genes selected through the DH procedure, such as embryo development, response to dicamba, or response to tissue culture, or perhaps the skewing is a random effect.

The chromosome arm 6AL region carrying *Sr67* also has *Sr13*, multiple alleles of which encodes a coiled-coil nucleotide-binding leucine-rich repeats (CNL) (Gill et al. 2021; Zhang et al. 2017). The *Sr13* gene-based marker Sr13F/R Primer (Zhang et al. 2017) was mapped in the RL6071 × KU168-2 DH population as a dominant marker and its coordinates on the IWGSC ReqSeq v2.0 are between 618,031,153–618033110 Mb (Fig. 1). The KASP marker *Kwh294* associated with *Sr67* mapped to a more proximal 616,436,883–616,436,975 Mb region, but suggesting a similar location of *Sr13* and *Sr67*. To date, four resistant alleles of *Sr13* (a–d) have been reported (Gill et al. 2021; Zhang et al. 2017). Given that KU168-2 failed to amplify markers (null alleles) associated with the *Sr13* alleles (Figs. 3, 4) and that sequencing primers used to amplify the *Sr13* CNL region also failed to amplify KU168-2 we propose that *Sr67* is located at a different locus (Fig. 5)*.*

In addition to the genotypic results, a high immune response to the multi-races as compared to the reference lines carrying *Sr13* alleles (a–d) clearly showed that *Sr67* is phenotypically unique compared to *Sr13* alleles (Fig. 2, Table 4). *Pgt* race RCRSF is a good example where phenotypic difference was demonstrated as this race was virulent to lines carrying *Sr13* alleles, whereas DH lines carrying *Sr67* were resistant. Moreover, all the *Sr13* alleles have tetraploid origin, whereas *Sr67* was discovered in a hexaploid accession. *Sr13* is one of the most important genes for stem rust resistance in durum wheat and lines with its different alleles produce low infection type in the range 2 to 3- (Gill et al. 2021), depending on ploidy and temperature with infection types being lower at higher temperatures (Roelfs and McVey 1979; Zhang et al. 2017), though it is known to be less effective in the hexaploid background, whereas *Sr67* has a more resistant response. Previously, *Sr13* was reported as showing a lower IT at higher temperatures, which was also observed in the present study (Table 4). In contrast, lines with *Sr67* generally showed a more variable low IT (see Supplemental Fig. 1) that was more effective at lower temperatures. Taken together, the phenotypic uniqueness, origin, and the finding that KU168-2 has null alleles for all molecular markers based on *Sr13* alleles provides compelling evidence that KU168-2 carries a resistance allele at a locus closely-linked to *SR13*.

Genetic resources, including effective seedling (all-stage) resistance genes, race-nonspecific adult-plant resistance, and DNA markers for marker-assisted selection of gene combinations, are important for developing new cultivars with durable resistance to stem rust. New stem rust resistance genes derived from the primary gene pool of wheat are particularly valuable resources, and thus, responsible deployment of these genes in combination will prolong their period of usefulness (durability). Here, we reported a new stem rust resistance gene that was discovered in a hexaploid wheat accession and developed KASP markers that can be used to select the gene in breeding and pre-breeding applications. *Sr67* and its markers represent new tools that can be utilized in breeding programs aimed at achievement of durable stem rust resistance.

### Supplementary Information

Below is the link to the electronic supplementary material.

Supplementary file1 (DOCX 808 kb)

## Data Availability

The datasets generated during the current study are available from the corresponding author on reasonable request.
