# Supplementary material for: Identification of Sr67, a new gene for stem rust resistance in KU168-2 located close to the Sr13 locus in wheat
Source: Theor Appl Genet. 2024 Jan 24;137(1):30. doi: 10.1007/s00122-023-04530-8 (PMC10808535; doi:10.1007/s00122-023-04530-8)
Supplement: Supplementary file 1 — Supplementary file1 (DOCX 808 kb) [file 122_2023_4530_MOESM1_ESM.docx]

**
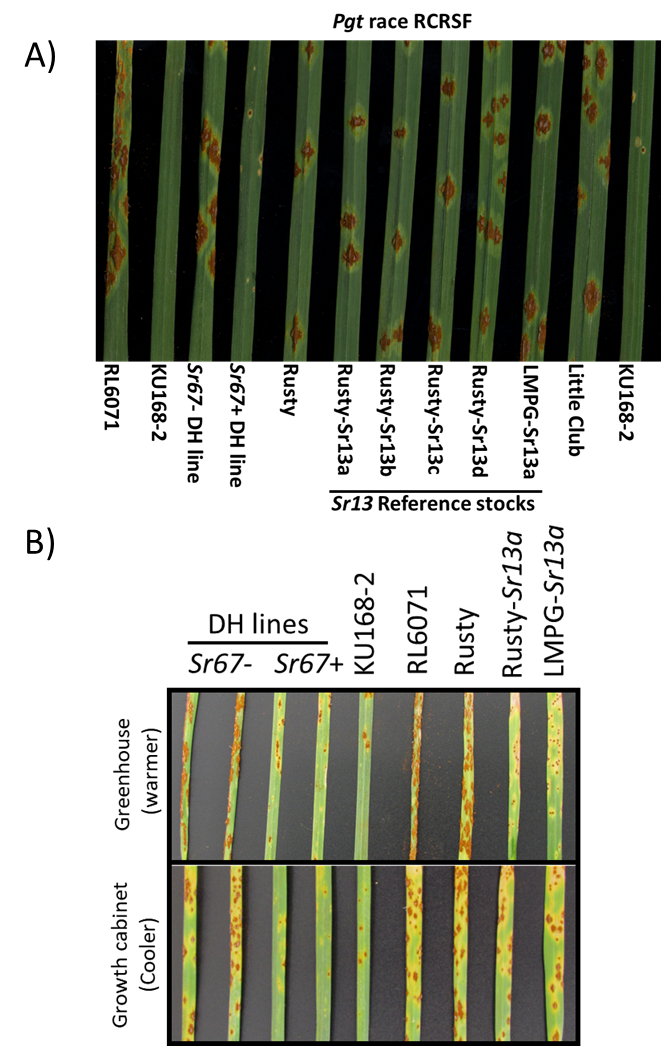
**

**Supplemental Figure 1: A) Comparison of seedling infection types of *Sr67* and *Sr13* alleles using *Pgt* race RCRSF. B) Comparison of *Sr67* with *Sr13* at lower and higher temperatures using *Pgt* race TPMKC.**
